# Gross Cystic Disease Fluid Protein-15 (GCDFP-15) Expression Characterizes Breast Mucinous Carcinomas in Older Women

**DOI:** 10.3390/diagnostics12123129

**Published:** 2022-12-12

**Authors:** Mayumi Kinoshita, Motoji Sawabe, Yurie Soejima, Makiko Naka Mieno, Tomio Arai, Naoko Honma

**Affiliations:** 1 Department of Molecular Pathology, Graduate School of Medical and Dental Sciences, Tokyo Medical and Dental University, Tokyo 113-8510, Japan; 2 Department of Clinical Laboratory Medicine, Faculty of Health Science Technology, Bunkyo Gakuin University, Tokyo 113-8668, Japan; 3 Department of Diagnostic Pathology, Hitachi General Hospital, Hitachi 317-0077, Japan; 4 Department of Medical Informatics, Center for Information, Jichi Medical University, Shimotsuke 329-0498, Japan; 5 Department of Pathology, Tokyo Metropolitan Geriatric Hospital, Tokyo 173-0015, Japan; 6 Department of Pathology, Faculty of Medicine, Toho University, Tokyo 143-8540, Japan

**Keywords:** breast, mucinous carcinoma, gross cystic disease fluid protein-15 (GCDFP-15), older, apocrine

## Abstract

The predominant histological subtype of breast mucinous carcinoma in older women is type B (hypercellular type), and, in younger women, it is type A (hypocellular type). The characteristics of mucinous carcinomas of the same histological subtype may differ between older and younger women. This study aims to systematically clarify the pathological/immunohistochemical features of mucinous carcinomas. A total of 21 surgical cases of mucinous carcinoma (type A/B: 9/12 cases) in the older group (≥65 years) and 16 cases (type A/B: 14/2 cases) in the younger group (≤55 years) (n = 37) were included. Gross cystic disease fluid protein-15 (GCDFP-15) and eight other markers were used for immunostaining. The GCDFP-15-positive rate in the older group was high regardless of the histological subtype (type A, 77.8%; type B, 91.7%). The GCDFP-15 positivity in the older group was significantly higher than that in the younger group (*p* < 0.001 for Allred score). Among type A, GCDFP-15 positivity was significantly higher in the older group than in the younger group (*p* = 0.042 for the Allred score and *p* = 0.007 for the positivity rate). The present results suggest that GCDFP-15 expression characterizes mucinous carcinomas in older women.

## 1. Introduction

Mucinous carcinoma is an invasive breast cancer histologically characterized by clusters of tumor cells suspended in extracellular mucin. Pure mucinous carcinoma is more common in older individuals and is generally associated with an excellent prognosis. Of all breast cancers, 2–4% are mucinous carcinomas, while in older women mucinous carcinomas account for more than 10% of breast cancer cases [1,2,3,4]. Histologically, pure mucinous carcinoma is classified as either type A (hypocellular: tubular, ribbon-like, and small papillary clusters with a large amount of extracellular mucin) or type B (hypercellular: large epithelial clumps or sheets with a small amount of extracellular mucin) [4,5]. Type B lesions are frequently positive for neuroendocrine markers (e.g., synaptophysin, chromogranin A, CD56) [6,7], and the surrounding tissues often have ductal carcinoma in situ (DCIS) components with a neuroendocrine tendency, which are considered precursor lesions [8].

In our previous study, approximately 16% of patients aged 85 years and older had mucinous carcinomas, and 11% had apocrine carcinomas [3]. In older patients with mucinous carcinomas, type B was predominant, as previously mentioned, and most type B lesions were positive for the apocrine marker gross cystic disease fluid protein-15 (GCDFP-15) [3,7]. Eosinophilic cytoplasm is also a characteristic cytological feature of type B lesions, which may reflect an apocrine character. Many lobular carcinomas in older patients are pleomorphic lobular carcinomas, which are referred to as apocrine-type lobular carcinomas. Type B mucinous carcinomas may also be considered apocrine-type mucinous carcinomas. For apocrine differentiated carcinomas, treatments targeting the androgen receptor (AR) have recently attracted attention due to their characteristic AR expression.

Mucinous carcinomas are generally considered hormone receptor-positive and human epidermal growth factor receptor 2 (HER2)-negative. They are the so-called luminal A-type cancers that have a favorable prognosis and a uniform clinical response [9,10]. However, genetic analysis has shown that type B mucinous carcinoma and neuroendocrine cancers have a common spectrum and a worse prognosis than type A [2]. Thus, the optimal clinical response may vary according to type A or B [11].

Type A and B mucinous carcinomas of the breast are common in younger and older women, respectively. As the hormonal environment of women varies greatly with age, even mucinous carcinomas of the same histological type may differ in their biological properties between older and younger individuals. In our previous study of mucinous carcinoma in older patients, there were special type A carcinomas with cytological features of type B carcinomas [7]. Therefore, this study aims to systematically clarify the clinicopathological features of mucinous carcinomas in older women by comparing the classical factors, such as type A/B, estrogen receptor (ER), progesterone receptor (PgR), HER2, nuclear grade, and Ki-67 score, as well as the expression of neuroendocrine and apocrine markers, with those from young to middle-aged women.

## 2. Materials and Methods

### 2.1. Subjects and Classification of Mucinous Carcinoma

The histological classification was based on the WHO classification [4]. Of the patients with surgical specimens diagnosed as pure-type mucinous carcinoma of the female breast between 2004 and 2017, 40 patients were aged 65 years and older (older group), and 16 were patients aged 55 years and younger (younger group).

Capella et al. reported that type A (hypocellular variant) has a mucus component of 60–90% while type B (hypercellular variant) has a mucus component of 33–75% [5]. In our study, to classify types A and B, specimens were evaluated by two separate pathologists. Specimen scores were obtained by scoring the cell component ratio using five levels (1: <20%, 2: 20–39%, 3: 40–59%, 4: 60–79%, 5: ≥80%), and cell cluster size was obtained by using three levels (1: small, 2: intermediate, and 3: large) and adding them [5]. Specimens with scores 2–3 and 6–8 were concordant, and they were classified as type A and type B, respectively. Cases with scores of 4 and 5 were evaluated as borderline, and when the outcome was discordant, the two pathologists reviewed the glass slides and decided together.

According to the histological type, there were 9 cases of type A and 31 cases of type B in the older group and 14 cases of type A and 2 cases of type B in the younger group (Fisher‘s exact test, *p* < 0.001). In the histological/immunohistochemical examination, 12 out of 31 cases of type B in the older group were available.

### 2.2. Clinicopathological Analysis

Pathological staging was based on UICC [12]. Nuclear grading was assessed according to the nuclear grading classification in the “Japanese Classification of Breast Cancer”, which is routinely used in Japan and has been confirmed to reflect the prognosis of Japanese breast cancer patients [13,14]. Briefly, the sum of nuclear atypia (1, mild; 2, moderate; 3, severe) and mitotic counts per 10 high-power fields (1, <5; 2, 5–10; 3, >10) was classified into a nuclear grade (I, 2 or 3; II, 4; III, 5 or 6).

### 2.3. Immunohistochemical Procedures and Evaluations

Immunohistochemical analyses for GCDFP-15, chromogranin A (CGA), synaptophysin (SYP), CD56, AR, ER, PgR, HER2, and Ki-67 were applied to the representative slides of formalin-fixed and paraffin-embedded tissues (Table 1). After antigen retrieval (none or heat-treatment for 40 min at pH6 or pH9), the slides were incubated for 30 min with the primary antibodies GCDFP-15, CGA, SYP, CD56, AR, ER, PgR, and Ki-67. After the endogenous peroxidase was quenched with 3% H_2_O_2_ in distilled water, the slides were incubated with secondary antibodies and detected using Histofine Simple Stain MAX-PO (MULTI) (Nichirei Biosciences Inc., Tokyo, Japan) and DAB substrate kits (Nichirei). HER2 immunohistochemical staining was performed according to the kit’s protocol (SV2-61γ, monoclonal: Nichirei).

To assess the staining, the percentage of immunoreactive cancerous cells was independently estimated in the nucleus (AR, ER, PgR, and Ki-67), cytoplasm (GCDFP-15, CGA, SYP, and CD56), and cytoplasmic membrane (HER2). We used the classification score proposed by Allred et al. for ER/PgR estimation in 1998 [15,16]. A positive case was defined as having an Allred score of 3 or more for CGA, CD56, AR, ER, and PgR or having a score of 4 or more for GCDFP-15 and SYP. In terms of HER2, a score of 3 was considered positive [17]. The Ki-67 score was defined as low when Ki-67-positive cells were <5% and high when Ki-67-positive cells were ≥5%. A high Ki-67 score was considered positive (Table 1).

### 2.4. Statistical Analyses

The Wilcoxon rank sum test was used to compare the Allred scores for each factor between the two groups. The Kruskal–Wallis test was used to compare the Allred score for each factor among the three groups. The Dunn test was used for pair-by-pair comparisons if the Kruskal–Wallis test was significant. Fisher’s exact test was used for contingency tables. The level of significance was set at *p* < 0.05. SPSS Statistics version 25 (IBM, Japan, Ltd., Tokyo, Japan) was used for statistical calculations.

## 3. Results

### 3.1. Clinicopathological Features

The mean age of the patients was 81.7 ± 6.8 years (range, 67–92 years) for the older group and 44.6 ± 8.6 years (range, 28–55 years) for the younger group. The T category that accounted for 50% or more of the cases was T2 for the older group and T1 for the younger group. The older group tended to have larger tumor sizes than the younger group. There were no N2 and N3 N-stage cases, and there was no significant difference between the two groups. All patients were negative for distant metastases. The TNM stage that accounted for 50% or more of the cases was stage II for the older group and stage I for the younger group. Nuclear grading showed no significant differences (Table 2). All patients were free from recurrence.

### 3.2. Immunohistochemical Study

**Figure 1:**
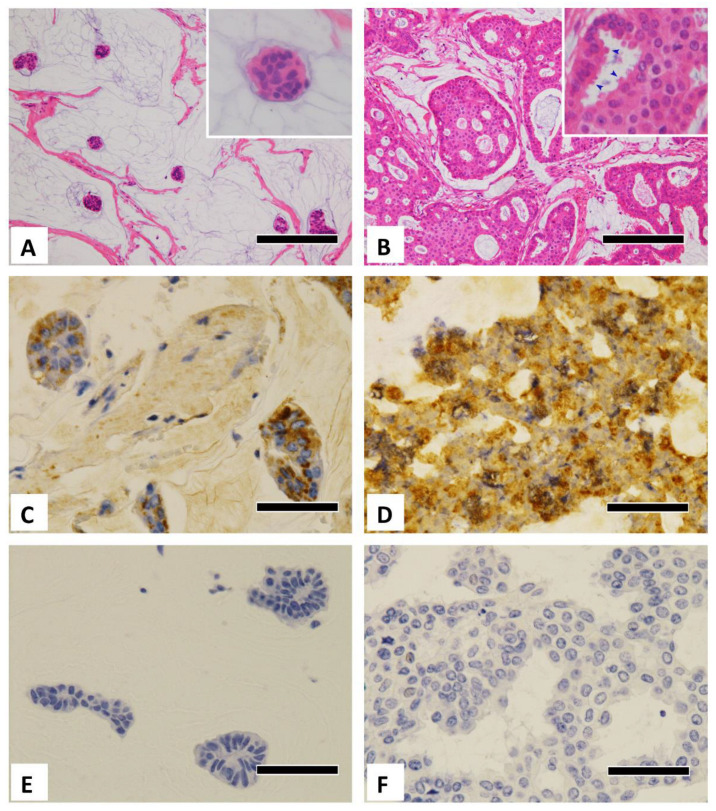
Microscopic pictures of breast mucinous carcinoma. (**A**) Histological image of a type A mucinous carcinoma. Note tubular and papillary small clusters with a large amount of extracellular mucin (HE stain). The inset shows a magnified image. (**B**) Histological image of a type B mucinous carcinoma. Large epithelial clumps or sheets composed of tumor cells with eosinophilic cytoplasm and a small amount of extracellular mucin (HE staining). The magnified image inset shows tumor cells with apocrine snouts (arrowheads) and abundant eosinophilic cytoplasm. (**C**) Older, type A (immunohistochemical pictures of GCDFP-15 positivity). (**D**) Older, type B (immunohistochemical pictures of GCDFP-15 positivity). (**E**) Younger, type A (immunohistochemical pictures of GCDFP-15 negativity). (**F**) Younger, type B (immunohistochemical pictures of GCDFP-15 negativity). GCDFP-15: gross cystic disease fluid protein-15. Scale bar = 200 μm (**A**,**B**), 50 μm (**C**–**F**).

**Figure 2:**
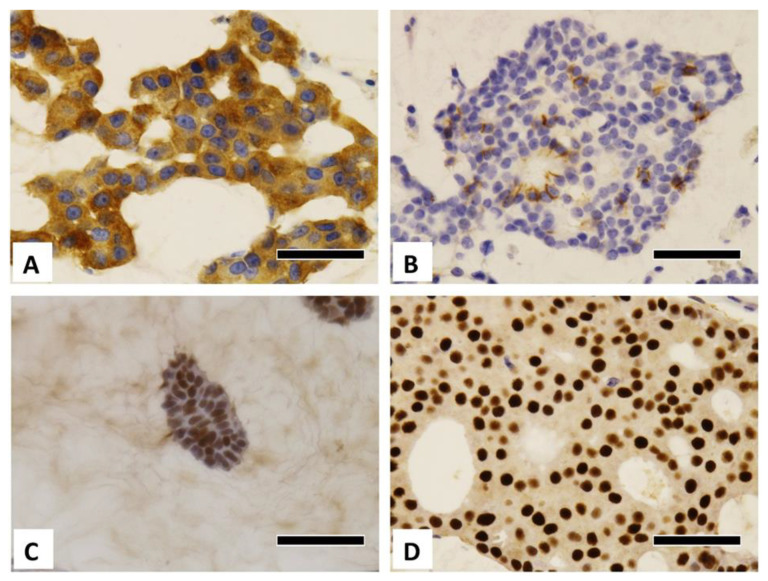
Immunohistochemical pictures of breast mucinous carcinoma. (**A**) SYP-positive cancer (older, type B), (**B**) CD56-positive cancer (older, type B), (**C**) AR-positive cancer (younger, type A), (**D**) AR-positive cancer (older, type B). AR, androgen receptor; SYP, synaptophysin. Scale bar = 50 μm.

Typical histological images of each type are shown in Figure 1A,B. Typical microscopic images of GCDFP-15 immunostaining from the different age groups and carcinoma types are shown in Figure 1C–F. Additional positive immunostaining images are shown in Figure 2. The Allred scores are statistically analyzed in Table 3, Table 4 and Table 5. When the cases were divided into four groups according to age group and carcinoma type, there were only two type B cases in the younger group. Thus, the remaining three groups were compared, as shown in Table 4 and Table 5.

#### 3.2.1. GCDFP-15

The Allred scores were significantly higher in the older group than in the younger group (*p* < 0.001), and they were significantly higher in type B carcinoma than in type A (*p* = 0.014) (Table 3). They were also significantly different among the three groups (*p* < 0.001), as shown in Table 4. No significant difference was observed between the older type A and older type B groups (*p* = 1.000), whereas a significant difference was observed between the older type A and younger type A groups (*p* = 0.042). In the dichotomous positive/negative comparison, GCDFP-15-positive expression was seen in 18 of 21 cases in the older group (85.7%) and 2 of 16 cases in the younger group (12.5%), yielding significant differences (*p* < 0.001). In the older group, 7 of 9 type A cases (77.8%) and 11 of 12 type B cases (91.7%) were positive for GCDFP-15, indicating a high positivity rate regardless of the carcinoma type (*p* = 0.553, Table 5. Figure 1C–F). Among type A, the positivity rate was significantly higher in the older group than in the younger group (*p* = 0.007).

#### 3.2.2. Neuroendocrine Markers

The Allred score for CGA was lower in the older group than in the younger group (*p* = 0.046) (Table 3), however, other CGA tests showed no significant differences (Table 4 and Table 5).

The Allred scores for SYP were insignificantly higher in the older group than in the younger group (*p* = 0.059) (Table 3). The difference was significant among the three groups (*p* = 0.024) (Table 4), however, no significant difference was obtained by pair-by-pair comparisons. The dichotomous analyses for SYP did not yield significant differences (Table 5).

CD56 did not differ significantly in any comparison (Table 3, Table 4 and Table 5).

#### 3.2.3. Steroid Hormone Receptors

There were no significant differences in the AR Allred scores between the older and younger patients (*p* = 0.250) or between type A and type B (*p* = 0.652) (Table 3) and in any further analyses (Table 4 and Table 5).

The ER Allred scores did not significantly differ between older and younger patients (*p* = 0.906) but were significantly higher in type B carcinoma than in type A (*p* = 0.032) (Table 3). No significant differences were found in further studies (Table 4 and Table 5). The PgR Allred scores did not differ significantly in any studies (Table 3, Table 4 and Table 5).

#### 3.2.4. HER2 and Ki-67 Immunostaining

There were no significant differences in the expression of HER2 and Ki-67 in any comparison (Table 3, Table 4 and Table 5).

#### 3.2.5. Comparison between GCDFP-15 Expression and Other Factors

**Figure 3:**
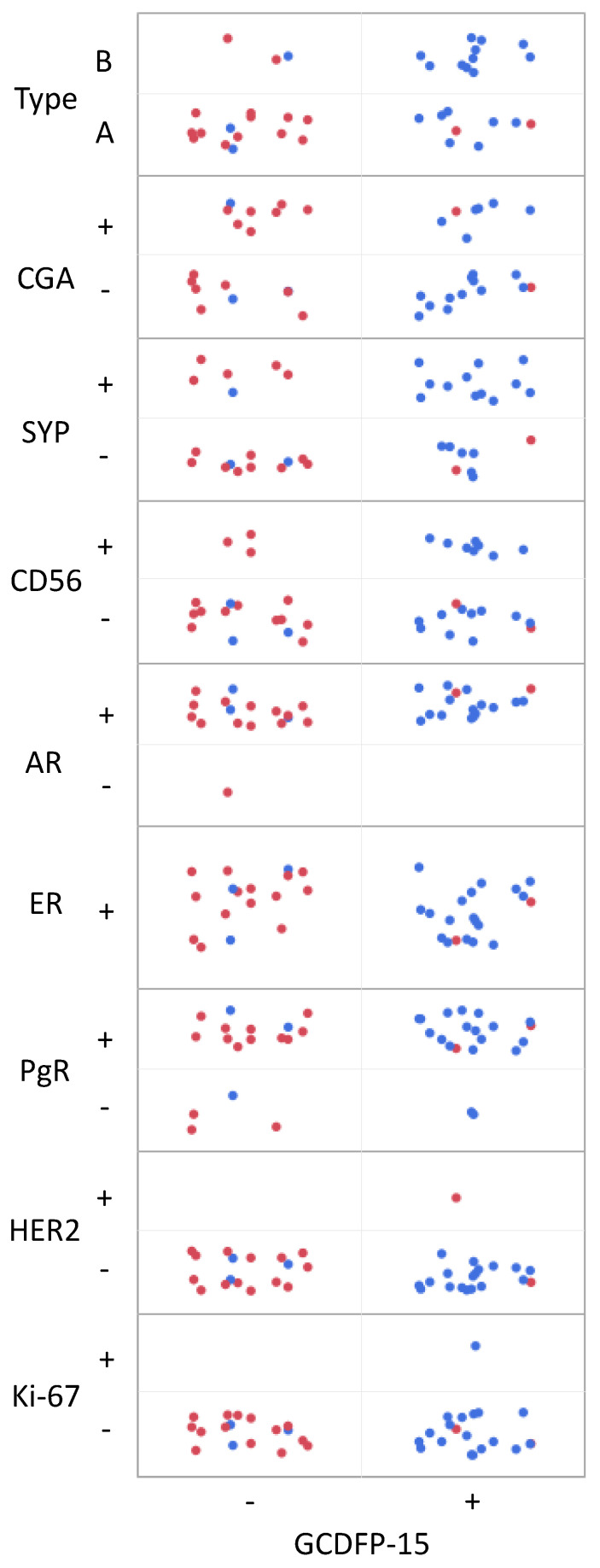
The relationships between GCDFP-15 expression and type of mucinous carcinoma or the expression of other immunohistochemical markers considering age (older patients in blue and younger patients in red). AR: androgen receptor; CGA: chromogranin A; ER: estrogen receptor; GCDFP-15: gross cystic disease fluid protein-15; HER2: human epidermal growth receptor 2; PgR: progesterone receptor; SYP: synaptophysin.

Figure 3 shows the relationships between GCDFP-15 expression and the type of mucinous carcinoma or the expression of other immunohistochemical markers considering age. Most of the mucinous carcinomas in older patients were GCDFP-15-positive irrespective of other factors, whereas those in younger patients exhibited opposite results.

## 4. Discussion

Our results showed that GCDFP-15 expression clearly characterizes mucinous carcinoma in older patients regardless of the mucinous carcinoma subtype or the other immunohistochemical markers. The neuroendocrine character was not necessarily characteristic of mucinous carcinoma of type B or in older patients.

### 4.1. Apocrine Markers (GCDFP-15/AR)

We previously reported, in older patients, a high rate of mucinous and apocrine cancers and higher rates of GCDFP-15 and AR-positive cancers [3]. In the present study, mucinous carcinomas in older patients were mostly positive for both GCDFP-15 and AR and showed abundant eosinophilic cytoplasm or apocrine snouts, suggesting their apocrine-like characteristics (Figure 1). Although apocrine metaplasia in mucinous carcinomas is described in the WHO classification [18], we clearly showed for the first time that GCDFP-15 positivity was more prevalent in older patients. So far, mucinous carcinoma in older patients has been characterized by type B morphology or neuroendocrine features; however, our results demonstrated that GCDFP-15 expression most clearly characterizes the mucinous carcinoma of older patients. Pleomorphic invasive lobular carcinoma, often GCDFP-15/AR-positive and regarded as an apocrine-type invasive lobular carcinoma, is frequent in older women [18,19,20,21]. As for tumors in the other organs, about 5% of lung adenocarcinoma were reported to be positive for GCDFP-15, and most of them occurred in older individuals [22,23]. Of note, GCDFP-15 has been reportedly positive for a mucin-rich variant of salivary duct carcinoma [24] and endocrine mucin-producing sweat gland carcinoma [25], both of which commonly affects older patients, suggesting a similar phenomenon in the other organs.

Interestingly, the type A mucinous carcinomas of older individuals also exhibited apocrine-like immunohistochemical characteristics (GCDFP-15 positivity and AR positivity). The relationship between type B carcinomas and neuroendocrine characteristics is well known. However, the apocrine-like characteristic was not limited to type B carcinomas, as de Andrade Natal reported [6] but rather was found in either type of mucinous carcinoma in older patients. Conversely, type A carcinomas in older patients might differ in their biological characteristics from type A carcinomas in younger patients. We conclude that the biologically essential features of mucinous carcinomas in older patients are apocrine-like immunohistochemical features (GCDFP-15/AR positivity), rather than neuroendocrine features. Of note, apocrine differentiation is generally characterized by GCDFP-15 positivity, AR positivity, ER negativity, and PgR negativity [18]. Almost all intraductal and invasive apocrine carcinomas are positive for GCDFP-15/AR and negative for ER and PgR [26]. Most mucinous carcinomas are ER-positive and PgR-positive, regardless of patient age and histological type, and thus they are not entirely apocrine-differentiated carcinomas— they partially have apocrine character. Interestingly, GCDFP-15 tended to be negative in younger patients despite AR positivity. These points are discussed further below.

### 4.2. GCDFP-15 and AR/PgR Expression

The expression of GCDFP-15 is induced by AR activation caused by the binding of androgens, such as testosterone or dihydrotestosterone, to AR [27]. In our study, there was no significant difference in the expression of AR between older and younger patients. Frequent expression of AR in mucinous carcinomas was previously reported; de Andrade Natal et al. reported that AR positivity was seen in 5 of 16 cases (31.6%) of type A breast mucinous carcinomas and 13 of 23 cases (56.5%) of type B breast mucinous carcinomas [6]. Cho et al. reported that the rate of AR positivity was 21.7% in breast mucinous carcinomas, of which 47.8% of all patients were 50 years old or older [28]. AR positivity is generally higher in luminal cancers (ER/PgR-positive cancers), and it may be reasonable that mucinous cancers with higher ER/PgR positivity have higher AR positivity. However, it is worth noting that, unlike in older patients, GCDFP-15 positivity was low in younger patients despite high AR positivity. As AR is structurally similar to PgR, progesterone has the ability to bind AR and inhibits its action [29,30]. AR action may be inhibited in younger patients due to their higher blood progesterone levels. The reduced GCDFP-15 expression in younger women might be a result of progesterone binding to the AR. Blood androgen levels decrease with age, albeit at a slow rate, whereas progesterone levels decrease sharply after menopause [31,32,33,34]. In older individuals, the androgen/progesterone ratio is higher than that in younger individuals. AR is less inhibited in the older groups, and this may maintain the GCDFP-15 expression. Consequently, GCDFP-15 positivity may have been higher in older patients than in younger patients. GCDFP-15 can be an indicator of normal androgen-AR signaling, as PgR is for ER; then, it may be revealed to work as a predictor of AR-targeting therapy in the future.

### 4.3. Expression of Other Immunohistochemical Markers

Previous reports showed a neuroendocrine feature in type B mucinous carcinomas [6,7,35]. Our results showed that SYP positivity in older women tended to be higher than that in younger individuals (Table 3), and that it was different among three groups (*p* = 0.024) (Table 4). In contrast, the CGA positivity was significantly higher in younger women than in older women and was not significantly different between type A and type B (Table 3) or in any other comparisons (Table 4 and Table 5). CD56 did not significantly differ in any comparison. Both SYP and CGA are good neuroendocrine markers with high sensitivity and specificity; however, the results of these neuroendocrine markers were inconsistent. Neuroendocrine features can also be examined by an electron microscope. Previous studies on mucinous carcinoma showed controversial results regarding the presence of neuroendocrine granules, suggesting “pseudo” neuroendocrine differentiation [36,37]. Thus, further studies are warranted to elucidate this neuroendocrine marker discrepancy.

Our immunohistochemical findings for ER, PgR, HER2, and Ki-67 suggested mucinous carcinomas were nearly all luminal A. A summary of previous reports is presented in Table 6. In all studies, more than 90% were positive for ER, and more than 80% were positive for PgR in most studies. The HER2 positivity rate was also low [1,6,38,39]. Our results were almost consistent with those reports regarding ER, PgR, and HER2. It is difficult to compare the results of Ki-67 as the Ki-67 index threshold is not universally standardized.

### 4.4. Limitations of This Study

The small sample size of our study necessitates studies with larger sample sizes to validate our results.

## 5. Conclusions

Our results showed that mucinous carcinomas in older patients are more clearly characterized by GCDFP-15 expression than type B or neuroendocrine differentiation, which has been considered to characterize them.

## Figures and Tables

**Table 1:** Experimental conditions for immunohistochemistry of breast mucinous carcinoma.

Primary Antibody	Primary Ab (Clone Name)	Dilution	Antigen Retrieval Method	Intracellular Localization	Positive Thresholds	Supplier
GCDFP-15	M (D6)	1:700	None	Cp	AS ≥ 4	SIGNET
CGA	P	RtoU	None	Cp	AS ≥ 3	Nichirei
SYP	M (27G12)	RtoU	None	Cp	AS ≥ 4	Nichirei
CD56	M (MRQ-42)	RtoU	40 min, pH 9	Cm	AS ≥ 3	Nichirei
AR	M (AR27)	1:25	40 min, pH 9	N	AS ≥ 3	Novocastra
ER	M (SPI)	RtoU	40 min, pH 9	N	AS ≥ 3	Nichirei
PgR	M (A9621A)	RtoU	40 min, pH 9	N	AS ≥ 3	Nichirei
HER2	M (SV2-61γ)	Kit	None	Cm	HS ≥ 3+	Nichirei
Ki-67	M (MIB-1)	1:200	40 min, pH 6	N	LI ≥ 5%	Dako

AR: androgen receptor; CGA: chromogranin A; ER: estrogen receptor; GCDFP-15: gross cystic disease fluid protein-15; HER2: human epidermal growth receptor 2; PgR: progesterone receptor; SYP: synaptophysin; Ab, antibody; M, monoclonal; P, polyclonal; RtoU, ready to use; Cp, cytoplasm; Cm, cell membrane; N, nucleus; AS, Allred score (total score); HS, HER2 score; LI, labeling index; SIGNET, SIGNET Lab, Inc., Dedham, USA; Nichirei, Nichirei Biosciences Inc., Tokyo, Japan; Novocastra, Novocastra Lab, Ltd., Sheffield UK; Dako Japan Inc., Tokyo, Japan.

**Table 2:** Clinicopathological summary of breast mucinous carcinoma.

	Older Group (≥65 y/o)	Younger Group (<55 y/o)	Fisher’s Exact Test (*p*-Value)
Number of cases	21	16	
Age, mean ± SD (range)	81.7 ± 6.81 (67–92)	44.6 ± 8.63 (28–55)	
T category (%)			0.733
T0	0 (0%)	0 (0%)	
T1	7 (33.3%)	8 (50.0%)	
T2	11 (52.4%)	6 (37.5%)	
T3	3 (14.3%)	2 (12.5%)	
T4	0 (0%)	0 (0%)	
N stage			1.000
N0	18 (85.7%)	14 (87.5%)	
N1	3 (14.3%)	2 (12.5%)	
N2, N3	0 (0%)	0 (0%)	
M category			
M0	21 (100%)	16 (100%)	
M1	0 (0%)	0 (0%)	
TNM stage			0.364
Stage 0	0 (0%)	0 (0%)	
Stage I	7 (33.3%)	8 (50%)	
Stage II	13 (61.9%)	6 (37.5%)	
Stage III	1(4.8%)	2 (12.5%)	
Stage IV	0 (0%)	0 (0%)	
Nuclear grade			0.832
Grade I	4 (19.0%)	4 (25.0%)	
Grade II	10 (47.6%)	8 (50.0%)	
Grade III	7 (33.3%)	4 (25.0%)	

SD, standard deviation.

**Table 3:** Comparisons of immunohistochemical features of breast mucinous carcinoma by age group and type.

	Median Score (Range)			Median Score (Range)		
Antibodies	Older	Younger	*p*-Value (Older vs. Younger)	Type A	Type B	*p*-Value (Type A vs. B)
Number of cases	21	16		23	14	
GCDFP-15	5 (0–8)	0 (0–5)	**<0.001**	3 (0–8)	5.5 (0–8)	**0.014**
CGA	0 (0–6)	2.5 (0–7)	**0.046**	2 (0–6)	0 (2–8)	0.394
SYP	4 (0–8)	2 (0–8)	0.059	3 (0–8)	6 (0–8)	0.186
CD56	0 (0–7)	0 (0–3)	0.201	0 (0–6)	1 (0–7)	0.237
AR	6 (3–8)	6 (2–7)	0.250	6 (4–8)	6 (2–8)	0.652
ER	8 (6–8)	7.5 (4–8)	0.906	7 (4–8)	8 (7–8)	**0.032**
PgR	6 (2–8)	7 (0–8)	0.376	6 (2–8)	5.5 (0–8)	0.525
HER2	0 (0–2)	0 (0–3)	1.000	0 (0–3)	0 (0–2)	0.904
Ki-67	1.5 (1–30)	1.5 (0–15)	0.874	1.5 (0–10)	1.75 (1–30)	0.190

*p*-values are calculated by the Wilcoxon rank sum test. AR: androgen receptor; CGA: chromogranin A; ER: estrogen receptor; GCDFP-15: gross cystic disease fluid protein-15; HER2: human epidermal growth receptor 2; PgR: progesterone receptor; SYP: synaptophysin.

**Table 4:** Immunohistochemical features of breast mucinous carcinomas among four subgroups.

	Median Score (Range)					
Antibodies	Older Type A (1)	Older Type B (2)	Younger Type A (3)	Younger Type B	*p*-Value (1) (2) (3) (KW Test)	*p*-Value (1) vs. (2)/(1) vs. (3) (Dunn Test)
Number of cases	9	12	14	2		
GCDFP-15	5 (0–8)	6 (3–8)	0 (0–5)	0, 0	**<0.001**	1.000/**0.042**
CGA	0 (0–6)	0 (0–6)	2 (0–6)	3, 7	0.124	n.a.
SYP	4 (0–8)	5 (0–8)	2 (0–5)	6, 8	**0.024**	1.000/0.093
CD56	0 (0–6)	1 (0–7)	0 (0–6)	0, 3	0.545	n.a.
AR	7 (4–8)	6 (3–8)	6 (4–7)	7, 2	0.447	n.a.
ER	7 (6–8)	8 (7–8)	7.5 (4–8)	7, 8	0.077	n.a.
PgR	6 (2–8)	5.5 (2–8)	7 (2–8)	0, 8	0.793	n.a.
HER2	0 (0–1)	0 (0–2)	0 (0–3)	0, 0	0.738	n.a.
Ki-67	1.5 (1–5)	1.5 (1–30)	1.25 (0–10)	5, 15	0.248	n.a.

*p*-values are calculated by the Kruskal–Wallis test and Dunn test. AR: androgen receptor; CGA: chromogranin A; ER: estrogen receptor; GCDFP-15: gross cystic disease fluid protein-15; HER2: human epidermal growth receptor 2; KW test: Kruskal–Wallis test; PgR: progesterone receptor; SYP: synaptophysin; n.a.: not applicable.

**Table 5:** Immunohistochemical features of carcinomas among four subgroups.

Antibodies	Older Type A (1)	Older Type B (2)	Younger Type A (3)	Younger Type B	*p*-Value (1) vs. (2)/(1) vs. (3)
	n = 9 +/−	n = 12 +/−	n = 14 +/−	n = 2 +/−	
GCDFP-15	7/2	11/1	2/12	0/2	0.553/**0.007**
CGA	4/5	3/9	6/8	2/0	0.397/1.000
SYP	6/3	7/5	3/11	2/0	1.000/0.077
CD56	3/6	5/7	2/12	1/1	1.000/0.343
AR	8/0	10/0	14/0	1/1	1.000/1.000
ER	9/0	12/0	14/0	2/0	1.000/1.000
PgR	8/1	10/2	12/2	1/1	1.000/1.000
HER2	0/9	0/12	1/13	0/2	1.000/1.000
Ki-67	1/8	3/9	4/10	2/0	0.603/0.611

*p*-values were calculated using Fisher’s exact test. AR: androgen receptor; CGA: chromogranin A; ER: estrogen receptor; GCDFP-15: gross cystic disease fluid protein-15; HER2: human epidermal growth receptor 2; PgR: progesterone receptor; SYP: synaptophysin.

**Table 6:** Reported immunohistochemical property of breast mucinous carcinoma.

Study	Group	Number of Cases	Mean Age	ER	PgR	HER2	Ki-67	Ki-67 Threshold
Our study	Older (67–92 y/o)	21	81.7	100%	85.7%	0%	19%	5%
	Younger (28–55 y/o)	16	44.6	100%	81.3%	6.3%	37.5%	5%
Li et al. [1]	50–89 y/o	2730	n.a.	96%	83%	n.a.	n.a.	n.a.
	30–49 y/o	516	n.a.	91%	81%	n.a.	n.a.	n.a.
Di Saverio et al. [38]	25–85 y/o	11422	68.3	94.1%	81.5%	n.a.	n.a.	n.a.
de Andrade Natal et al. [6]	Type A	17	57.0	100%	52.9%	5.9%	0%	14%
	Type B	23	66.0	95.7%	73.9%	4.3%	21.7%	14%
Lacroix-Triki et al. [39]		35	n.a.	100%	85.7%	2.9%	8.6%	10%

ER, estrogen receptor; HER2, human epidermal growth receptor 2; PgR, progesterone receptor; n.a., not available.

## Data Availability

No applicable.
